# The Transcription Factors Tbx18 and Wt1 Control the Epicardial Epithelial-Mesenchymal Transition through Bi-Directional Regulation of Slug in Murine Primary Epicardial Cells

**DOI:** 10.1371/journal.pone.0057829

**Published:** 2013-02-28

**Authors:** Makiko Takeichi, Keisuke Nimura, Masaki Mori, Hironori Nakagami, Yasufumi Kaneda

**Affiliations:** 1 Division of Gene Therapy Science, Graduate School of Medicine, Osaka University, Suita, Osaka, Japan; 2 Stem Cell Program, Boston Children’s Hospital, Boston, Massachusetts, United States of America; 3 Division of Vascular Medicine and Epigenetics, United Graduate School of Child Development, Osaka University, Suita, Osaka, Japan; Tokai University, Japan

## Abstract

During cardiac development, a subpopulation of epicardial cells migrates into the heart as part of the epicardial epithelial-mesenchymal transition (EMT) and differentiates into smooth muscle cells and fibroblasts. However, the roles of transcription factors in the epicardial EMT are poorly understood. Here, we show that two transcription factors expressed in the developing epicardium, T-box18 (*Tbx18*) and Wilms’ tumor 1 homolog (*Wt1*), bi-directionally control the epicardial EMT through their effects on Slug expression in murine primary epicardial cells. Knockdown of Wt1 induced the epicardial EMT, which was accompanied by an increase in the migration and expression of N-cadherin and a decrease in the expression of ZO-1 as an epithelial marker. By contrast, knockdown of Tbx18 inhibited the mesenchymal transition induced by TGFβ1 treatment and Wt1 knockdown. The expression of Slug but not Snail decreased as a result of Tbx18 knockdown, but Slug expression increased following knockdown of Wt1. Knockdown of Slug also attenuated the epicardial EMT induced by TGFβ1 treatment and Wt1 knockdown. Furthermore, in normal murine mammary gland-C7 (NMuMG-C7) cells, Tbx18 acted to increase Slug expression, while Wt1 acted to decrease Slug expression. Chromatin immunoprecipitation and promoter assay revealed that Tbx18 and Wt1 directly bound to the *Slug* promoter region and regulated *Slug* expression. These results provide new insights into the regulatory mechanisms that control the epicardial EMT.

## Introduction

During cardiac development, cells derived from the proepicardium are distributed over the heart to form the epicardium, i.e., the outer cell layer, and these epicardial cells contribute to coronary vessel formation [1], [2]. A subpopulation of epicardial cells undergoes the epicardial epithelial-mesenchymal transition (EMT) to generate a population of mesenchymal cells that migrate into the underlying myocardium and give rise to fibroblasts and smooth muscle cells of the coronary arteries. A recent study demonstrated that epicardial cells expressing Tcf21 develop into cardiac fibroblasts and smooth muscle cells [3]. Two different lineage-tracing studies using Cre-LoxP technology (Tbx18-Cre or Wt1-Cre) have demonstrated that epicardial cells expressiong Tbx18 can differentiate into cardiomyocytes, coronary smooth muscle cells and fibroblasts [4], while Wt1-positive epicardial cells generate cardiomyocytes, smooth muscle cells and endothelial cells [5]. However, recent studies have disputed the myocardial and endothelial fates of epicardial cells in mice. Tbx18 is expressed in cardiomyocytes [6], [7], and Wt1 is expressed in endothelial cells [8]; therefore, Cre recombination occurs not only in the epicardium but also in other types of cells. Furthermore, Wt1-Cre recombination occurs ectopically with low efficiency [8]. In addition, it is widely accepted that vascular endothelial cells arise from venous cells but not from the epicardium [9]. Despite the debate regarding the fate of epicardial cells, the importance of the epicardium can be inferred from the fact that epicardial defects are embryonically lethal in various mouse models [2], [10]. Several molecules, including various soluble factors [2], [11] and Notch [12], [13], have been shown to be important for the epicardial EMT and differentiation. However, the roles of transcription factors in the epicardial EMT remain unclear.

The transcription factors Tbx18 and Wt1 are expressed in the proepicardium and embryonic epicardium. Tbx18 is expressed at high levels in embryonic tissues [14], and Wt1 is expressed in other mesothelia as well as in the epicardium and the developing genitourinary system [15]. During cardiogenesis, Tbx18 regulates myocardial differentiation [16], [17], although the epicardium develops normally in Tbx18-deficient mice. Transgenic mice that overexpress Tbx18 in epicardium-derived cells exhibit no defects in the differentiation and migratory behavior of epicardial cells [18]. Deletion of Wt1 causes embryonic lethality, peripheral edema, pericardial hemorrhage and thinning of the myocardial wall [19], [20]. Recent studies have demonstrated that Wt1 functions as a positive regulator of the epicardial EMT through the regulation of E-cadherin and Snail [21] or through retinoic acid signaling [22], [23] in the heart. By contrast, Bax et al. reported that the mesenchymal transition is induced by Wt1 knockdown in cultured human adult epicardial cells, indicating that Wt1 is necessary for maintaining the epicardial properties of cultured cells [24]. Because Wt1 is essential for the mesenchymal-epithelial transition (MET) of renal mesenchymal cells during kidney development [25], it has been suggested that Wt1 expression shifts the epithelial-mesenchymal balance [26].

Tbx18 and Wt1 are not expressed in the adult mouse epicardium; however, the expression of these molecules is upregulated after injury [27]–[29]. It has been suggested that adult mouse epicardial cells contribute to heart regeneration after injury by secreting paracrine factors [28] or differentiating into cardiomyocytes [29], although the myocardial differentiation of epicardial cells remains controversial [30], [31]. Because Tbx18 and Wt1 are expressed in epicardial cells with the potential for transformation during development and regeneration, it is possible that Tbx18 and Wt1 mediate the epicardial EMT in the embryonic or adult epicardium.

In this study, we used primary embryonic epicardial cells and investigated the roles of Tbx18 and Wt1 in the epicardial EMT, excluding the effects of these molecules in the early stages of heart development. Our data suggest that the epicardial transcription factors Tbx18 and Wt1 bi-directionally regulate Slug expression, which is important for the mesenchymal transition of epicardial cells.

## Materials and Methods

### Ethics Statement

This study was approved by the committee of the Institute of Experimental Animal Science, Osaka University Medical School (Number: J004548-011), and was conducted in accordance with institutional guidelines.

### Primary Culture of Epicardial Cells and Cardiomyocytes

Primary epicardial cells were prepared according to a previously described method with some modifications [32]–[34]. Hearts were dissected from E12.5 C57BL/6J mouse embryos and placed dorsal side-down in collagen I-coated 12-well dishes containing Dulbecco’s modified Eagle’s medium (DMEM, Nacalai Tesque), with 10% FBS. After an overnight incubation, the hearts were removed, and the medium was changed. The cobblestone-like epicardial cells that remained attached to the dishes were grown at 37°C until they could be used in experiments. Colonies containing too few primary epicardial cells were excluded from the analyses after heart removal. Primary cardiomyocytes were also prepared from the ventricles of E12.5 embryos, which were digested overnight with trypsin-EDTA, as has been described previously, with modifications [35]. Cardiac fibroblasts were removed by pre-plating the cells on collagen I-coated dishes for 1 hr.

Primary epicardial cells were cultured in 10% FBS/DMEM containing recombinant soluble factors, such as TGFβ1 (10 ng/ml; R&D systems), FGF2 (100 ng/ml; Roche), BMP4, BMP2, FGF9, VEGF, PDGF-BB (100 ng/ml; Peprotech) or retinoic acid (1 µmol/l; Sigma-Aldrich), for 3 days. The soluble factors were added on the day of heart removal. The medium was replaced daily. For western blot analysis, cells were treated with TGFβ1 for 2 days, beginning on the day following heart removal, at a final concentration of 1 ng/ml.

### Immunostaining of Primary Epicardial Cells

Primary epicardial cells were prepared by placing E12.5 hearts on collagen-coated cover glasses (Iwaki) and cultured as described above. The cells were then fixed with 4% paraformaldehyde (PFA) in PBS for 15 min and permeabilized with 0.2% Triton X-100 for 10 min at room temperature. Following a wash in PBS, the cells were blocked with a solution of 3% BSA in PBS for 1 hr at room temperature and incubated with primary antibodies directed against Wt1 (1∶50, sc-192; Santa Cruz Biotechnology), ZO-1 (1∶200, 61-7300; Invitrogen) or N-cadherin (1∶50, 610920; BD Transduction Laboratories) at 4°C overnight. The cells were then washed with PBS (3×10 min) and incubated with Alexa Fluor 488 anti-rabbit IgG (Invitrogen) or Alexa Fluor 488 anti-mouse IgG (Invitrogen) for 1 hr at room temperature. After washing with PBS (3×10 min), the cells were mounted with Prolong Gold anti-fade reagent containing DAPI (Invitrogen) and observed under a confocal laser microscope (Nikon).

### Knockdown Experiments in Primary Epicardial Cells

For knockdown experiments, stealth siRNA (Invitrogen) or control siRNA (Sigma-Aldrich) was transfected using RNAiMax (Invitrogen) on the day following heart removal. The following siRNAs were used: Tbx18 siRNA (siTbx18; MSS233533, siTbx18-2; MSS233532, Invitrogen), Wt1 siRNA (siWt1; MSS212627, siWt1-2; MSS212628, Invitrogen), Slug siRNA (siSlug; MSS237944, Invitrogen) and control siRNA (siControl; SIC-001, Sigma-Aldrich). Two days after transfection, either RNA or proteins were isolated from the cells. If both siRNA and TGFβ1 were used, TGFβ1 was added on the day following siRNA transfection. Primary cells were incubated with DMEM containing 10% FBS and 1 ng/ml TGFβ1 for 1 day prior to the isolation of RNA or protein.

### Wound Healing Assay in Primary Epicardial Cells

siRNA transfection was performed on the day following heart removal. Two days after transfection, the primary epicardial colonies were scratched [21]. The migration distance was calculated by measuring the distance between the cell walls at 0 hr and 11 hr after the scratch was made.

### BrdU Staining of Primary Epicardial Cells

siRNA transfection was performed on the day following heart removal. Two days after transfection, primary epicardial cells were incubated with 10% FBS/DMEM containing BrdU (Sigma-Aldrich, 50 µg/ml) at 37°C for 4 hr. After the cells were fixed and permeabilized as described above, the cells were incubated with 2 N HCl at 37°C for 1 hr. After washing with PBS, the cells were blocked with a solution of 3% BSA in PBS for 1 hr at room temperature and incubated with primary antibodies directed against BrdU (1∶200, ab6326; Abcam) at 4°C overnight. The cells were then washed with PBS (3×10 min) and incubated with Alexa Fluor 488 anti-rat IgG (Invitrogen) for 1 hr at room temperature. After washing with PBS (3×10 min), the cells were mounted and observed.

### Primary Epicardial Cell Count

Based on their cellular morphologies, we categorized the primary epicardial cells into two groups (“Enlarged” and “Cobblestone-like”). We measured the size of the non-treated epicardial cells with ImageJ and defined compact cobblestone-like cells smaller than 0.0025 mm^2^ as “Cobblestone-like.” These criteria accurately reflected the cellular morphology. The counted areas were determined such that the distance from the edge of each colony was 820 µm.

### RNA Isolation and Real-time PCR

Total RNA was isolated from primary epicardial cells and primary cardiomyocytes using Isogen (Nippon gene). Each RNA sample was prepared from 4 epicardial colonies. The RNeasy Mini Kit (Qiagen) was used to isolate RNA from NMuMG-C7 cells. In each experiment, cDNA was synthesized from an equal quantity of total RNA (400–800 ng for primary cells and 2 µg for NMuMG-C7 cells) by reverse transcription using the High Capacity RNA-to-cDNA Kit (Applied Biosystems). qPCR was performed with SYBR Premix Ex Taq (Takara) when using oligonucleotide primers (Table S1) or with the Realtime PCR Master Mix (Toyobo) when performing Taqman gene expression assays (Applied Biosystems, *Tbx5*; Mm00803518_m1, *Tbx18*; Mm00470177_m1, *Wt1*; Mm00460570_m1, *E-cadherin*; Mm00486918_m1, *ZO-1*; Mm00493699_m1, *Zyxin*; Mm00496120_m1 and *Gapdh*; Mm99999915_g1) with a C1000 thermal cycler (BioRad). Oligonucleotide primers were designed with the Universal ProbeLibrary Assay Design Center (Roche). The RNA was sequentially diluted with water to generate templates for the creation of a standard curve (1∶5 dilution, 5 templates). The mRNA level of each gene was calculated based on the standard curve using CFX manager software (BioRad) [36], [37]. The specific amplification of the cDNA was confirmed by the melt curve and the electrophoresis of the real-time PCR products. The quantified mRNA levels were normalized to *Gapdh* mRNA expression (as an internal control). Assays were performed in triplicate within a single experiment, and the average values were used for further analysis. Graphs, statistics and error bars are shown for 3 independent experiments.

### Western Blotting

Primary epicardial cells were scraped, washed once with PBS and dissolved in NuPAGE reducing sample buffer (Invitrogen). To denature the proteins, the samples were vortexed for 5 min and heated to 96°C for 5 min, and this was repeated 3 times. The loading volume was determined by normalizing the samples to the amount of Histone H3. Protein samples from 10–20 epicardial colonies were loaded in a single lane. NMuMG-C7 cells were washed twice with PBS and lysed with RIPA Buffer (Pierce). After centrifugation, the supernatants were quantified with the Bio-Rad DC Protein Assay (Bio-Rad), mixed with sample buffer (Bio-Rad) and heated to 96°C for 5 min to denature the proteins. SDS-PAGE and western blotting were performed as described previously [38]. The level of protein expression was analyzed using antibodies against Wt1 (1∶100, sc-192; Santa Cruz Biotechnology), Tbx18 (1∶100; sc-17869; Santa Cruz Biotechnology), E-cadherin (1∶2500; 610181; BD Transduction Laboratories), Vcam1 (1∶500; AF643; R&D Systems), N-cadherin (1∶5000; 610920; BD Transduction Laboratories), Snail (1∶200; 3895; Cell Signaling), Slug (1∶200; 9585; Cell Signaling) and ZO-1 (1∶250; 61–7300; Invitrogen). Anti-Histone H3 (1∶2000; ab1791; Abcam) and anti-β-actin (1∶100000; A5441; Sigma-Aldrich) antibodies were used as internal controls. All of the primary antibodies were detected with secondary antibodies conjugated to horseradish peroxidase (anti-mouse IgG, GE healthcare; anti-rabbit IgG, GE healthcare; anti-goat IgG, Jackson ImmunoResearch). For stripping, the membranes were incubated with WB Stripping Solution Strong (Nacalai Tesque) for 15 min at room temperature. All western blotting experiments were performed at least twice.

### NMuMG-C7 Cell Culture and Establishment of Stable Cell Lines

NMuMG cells were obtained from ATCC. As described previously [38], we cloned the cells by limiting dilution and obtained 13 different clones. From these colonies, we chose a cell line designated ‘C7,’ which exhibited a typical epithelial morphology and a robust response to TGFβ1. The NMuMG-C7 cells were maintained in DMEM containing 10% FBS and penicillin/streptomycin and were used to establish stable cell lines as previously reported [38]. Briefly, the mouse *Tbx18*, mouse *Wt1* and *EGFP* sequences were amplified by PCR, cloned into the pCX4 vector [39] and used for retroviral production. NMuMG-C7 cells were incubated in conditioned medium containing retrovirus and polybrene overnight. After the medium was changed, these cells were maintained in medium with antibiotics to select transduced cells. The antibiotics were removed during the subsequent experiments.

### Migration Assay of NMuMG-C7 Cells

Boyden’s chamber assay was performed as previously described [38]. DMEM containing 10% FBS was added to the lower chamber, and cells suspended in DMEM without FBS were added to the upper chamber. The membrane was incubated with a 0.1% gelatin solution for 1 hr at 37°C prior to use. After a 6-hr incubation, the cells on the lower side of the membrane were stained and counted.

### Native ChIP Assay

The native ChIP assay was performed as described previously [40]. A NMuMG-C7 cell line stably expressing 3×FLAG-tagged Tbx18 was established by electroporation, and the Tbx18 protein was immunoprecipitated with an anti-FLAG M2 affinity gel (A2220; Sigma-Aldrich) from the nuclear fraction of this stable cell line. Wt1 was immunoprecipitated from the nuclear fraction of a cell line that stably expresses Wt1 with an anti-Wt1 antibody (sc-192; Santa Cruz Biothechnology). Normal mouse (ab18413; Abcam) or rabbit (ab46540; Abcam) IgG was used as a control. DNA fragments that were co-precipitated with the Tbx18 or Wt1 proteins were amplified by real-time PCR with specific primer sets that targeted the *Slug* promoter region (Table S1), and the results were normalized to the amount of H1Foo DNA (Table S1).

### Promoter Assay

The mouse *Slug* promoter region (from −1550 to +1000) was generated by PCR and cloned into the pGL4-basic vector (Promega). siRNAs were transfected into primary epicardial cells as described above. On the day following siRNA transfection, the pGL4-*Slug*-promoter construct was transfected into primary epicardial cells with the Fugene HD transfection reagent (Promega). After 24 hr of incubation, the luciferase activity was measured with the Dual-Luciferase reporter assay system (Promega). Constructs of pGL4-*Slug* promoter lacking the putative Wt1- or Tbx18-binding regions (−200 del and +350 del) were generated by PCR. These plasmids were transfected 2 days after heart removal, and the luciferase activity was measured after 24 hr of incubation as described above.

### Statistics

Statistical analyses were performed with JMP 9 software (SAS Institute Inc.). All results were expressed as the mean ± SD. The data were compared using Tukey’s test. Significance was defined as *P*<0.05.

## Results

### Primary Culture of Epicardial Cells from E12.5 Mouse Embryos

**Figure 1:**
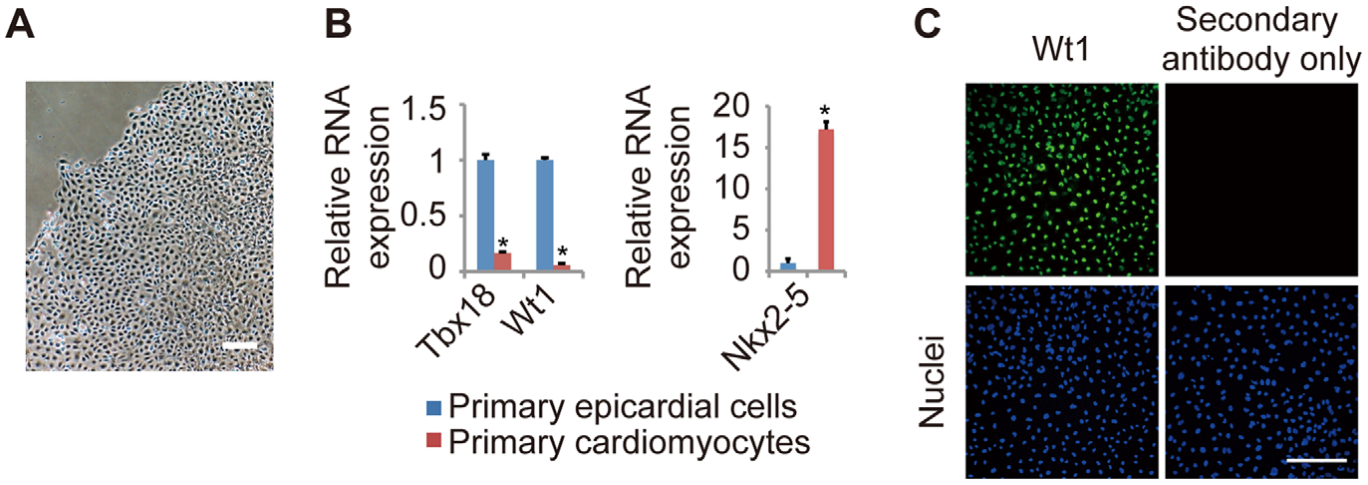
Primary culture of epicardial cells from E12.5 mouse embryos. (A) Representative image of primary epicardial cells generated from E12.5 mouse hearts, as described in the Materials and Methods section. (B) The relative mRNA expression levels of epicardial markers (*Tbx18 and Wt1*) and a cardiomyocyte marker (*Nkx2-5*) in primary epicardial cells and cardiomyocytes, as determined by quantitative real-time PCR (n = 3; *P<0.0001 vs. primary epicardial cells). The results are normalized to *Gapdh* expression, and the relative expression level is given as a ratio to primary epicardial cells. (C) Immunostaining for Wt1 (green) and DAPI nuclear staining (blue) of primary epicardial cells after 4 days in culture. The data are presented as the mean ± SD. Scale bars: 200 µm.

To investigate the molecular functions of Tbx18 and Wt1 in the epicardial EMT, we developed a primary epicardial cell culture from E12.5 mouse embryos. After 4 days of culture, these primary cells exhibited a cobblestone-like morphology (Figure 1A) and demonstrated high levels of mRNA expression of the epicardial markers *Wt1* and *Tbx18* but not the cardiomyocyte marker *Nkx2-5* (Figure 1B). The epicardial cells were confirmed to be 97% pure by immunofluorescent staining with an anti-Wt1 antibody (Figure 1C). These data indicate that primary embryonic epicardial cells can be successfully cultured with high purity.

### The Knockdown of Tbx18 and Wt1 Induces Distinct Effects in Primary Epicardial Cells

**Figure 2:**
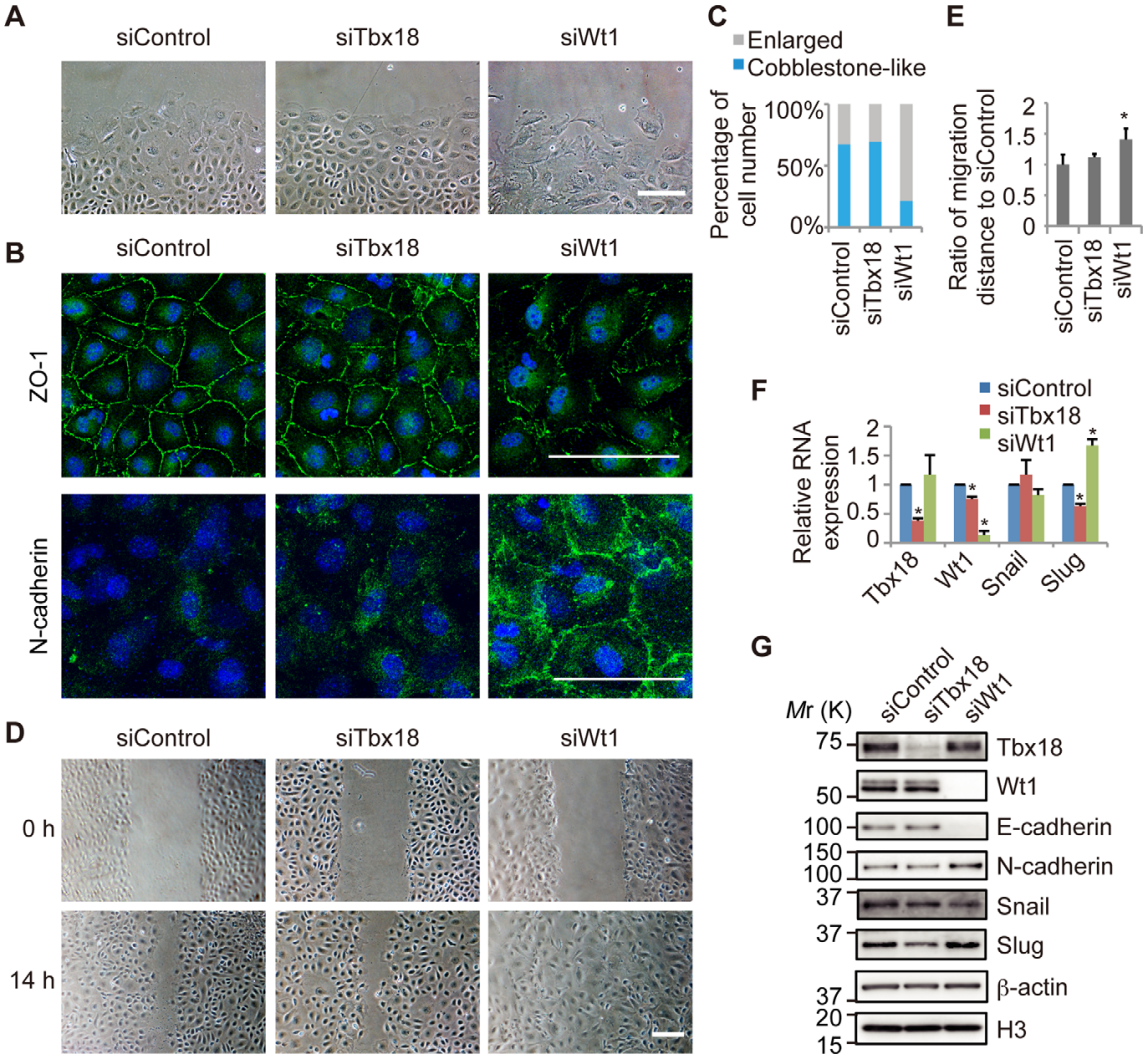
Knockdown of Wt1 and Tbx18 in primary epicardial cells. (A) Representative images of primary epicardial cells transfected with control siRNA (siControl) or siRNA directed against Tbx18 (siTbx18) or Wt1 (siWt1). Scale bar: 200 µm. (B) Immunostaining for ZO-1 or N-cadherin (green) and DAPI nuclear staining (blue) of primary epicardial cells transfected with siRNAs. Scale bars: 100 µm. (C) Percentage of cells categorized as “Enlarged” or “Cobblestone-like,” based on the cellular morphology of primary epicardial cells transfected with siRNAs. (D) Representative images of primary epicardial cells transfected with siRNAs at 0 and 14 hr after the scratch was made. Scale bar: 200 µm. (E) Quantification of migration distance, given as a ratio to the siControl (n = 4; *P<0.01 vs. siControl). (F) The relative mRNA expression of *Tbx18*, *Wt1*, *Snail* and *Slug* by real-time PCR analysis (n = 3; *P<0.05 vs. siControl). The results were normalized to *Gapdh* expression, and the relative expression level is given as a ratio to the siControl. (G) Western blot performed with antibodies against Tbx18, Wt1, adhesion molecules (E-cadherin and N-cadherin) and EMT regulators (Snail and Slug). β-actin and histone H3 were used as loading controls. The data are presented as the mean ± SD.

Tbx18 and Wt1 are transcription factors that are expressed in the embryonic epicardium. To investigate the functions of Tbx18 and Wt1 in the epicardial EMT, we performed knockdown experiments of Tbx18 and Wt1 in primary embryonic epicardial cells. Cells treated with control siRNA (siControl) maintained a cobblestone-like cell shape, although a slight morphological change was observed at the edge of the colony after several days in culture (Figure 2A). ZO-1, an epicardial and epithelial adhesion molecule, was localized at the cell-cell junction, and N-cadherin, a mesenchymal adhesion molecule, was not observed in the siControl cells (Figure 2B). Knockdown of Wt1 (siWt1) induced significant morphological changes, including impaired cell-cell contacts and a larger cell size (Figure 2A). In Wt1-knockdown cells, ZO-1 expression was decreased; however, N-cadherin was increased at the cell-cell interfaces, suggesting that the Wt1 knockdown induced the mesenchymal transition (Figure 2B). Knockdown of Tbx18 (siTbx18) did not affect cell-cell interactions or ZO-1 localization at the cell-cell junctions, and all cells, including those at the edge of the colony, maintained a more compact cell shape when compared to control cells (Figure 2A and 2B). Furthermore, knockdown of Wt1 but not Tbx18 significantly increased the proportion of enlarged cells (Figure 2C and Figure S1A).

Because cell motility is known to increase following the mesenchymal transition [41], cell migratory activity was examined using the scratch assay. After 14 hours, the scratch had completely closed (Figure 2D), and the migration distance was significantly increased in Wt1-knockdown cells but not in control or Tbx18-knockdown cells (Figure 2E). Furthermore, knockdown of Tbx18 or Wt1 did not affect cellular proliferation (Figure S2), indicating that Wt1 knockdown promoted the migration of primary epicardial cells.

The transcription factors Snail and Slug are expressed in epicardial cells [22] and are candidate regulators of the epicardial EMT. As shown in Figure 2F and 2G, the expression of both Slug mRNA and Slug protein was decreased in Tbx18-knockdown cells and increased in Wt1-knockdown cells, and these changes were significantly correlated with the observed changes in cellular morphology. However, there was no significant change in *Snail* mRNA expression following knockdown, suggesting that Snail is not a downstream target of either Tbx18 or Wt1 (Figure 2F). Moreover, expression of the Snail protein was slightly decreased in Wt1-knockdown cells, consistent with a recent report [21] but not with the observed cellular morphology (Figure 2G). These results were confirmed in another set of experiments with different siRNAs (siTbx18-2 and siWt1-2) (Figure S3).

Western blot analysis revealed that Wt1 knockdown resulted in a decrease in E-cadherin expression (Figure 2G); however, E-cadherin was not detected by immunofluorescence, suggesting that the expression level of E-cadherin is low and might not be essential for the epicardial properties. An increase in N-cadherin expression was also observed by western blot analysis as well as by immunofluorescent staining (Figure 2B and 2G). Based on these results, we hypothesized that knockdown of Wt1 may induce the epicardial EMT by upregulating Slug expression.

### Knockdown of Tbx18 or Slug Inhibits TGFβ1-induced Morphological Changes in Primary Epicardial Cells

To further assess the roles of Tbx18, Slug and Wt1 in the epicardial EMT, we performed knockdown experiments of these transcription factors during the epicardial EMT induced by TGFβ1. Embryonic epicardial cells are generated from the proepicardium and cover the surface of the heart; subsequently, they migrate into the heart and differentiate into other types of cells as part of the epicardial EMT. These processes are regulated by numerous soluble factors, including TGFβ1, BMP4, BMP2, FGF2, FGF9, VEGF, PDGF-BB and retinoic acid [2], [11], [42], [43].

To identify effective epicardial EMT inducers in the primary epicardial cells used in this study, we examined the ability of the soluble factors to induce the epicardial EMT. As shown in Figure S4A, untreated primary epicardial cells exhibited a cobblestone-like morphology and maintained cell-cell adhesion. By contrast, TGFβ1-treated cells and BMP4-treated cells adopted a spread shape, and the cells at the edge of the colony appeared scattered and larger in size, indicating that both TGFβ1 and BMP4 strongly induced the mesenchymal transition in these primary epicardial cells. Although treatment with other soluble factors (BMP2, FGF2, FGF9, VEGF, PDGF-BB and retinoic acid) slightly increased the number of scattered cells at the edges of the colonies, none of these factors led to significant morphological changes. To further assess the observed mesenchymal transition, we examined the expression of adhesion molecules. Treatment with either TGFβ1 or BMP4 resulted in a decrease in the expression of epicardial adhesion molecules such as *Integrin α4* and *Vcam1* and an increase in the expression of the mesenchymal adhesion molecule *N-cadherin* (Figure S4B). These results suggest that TGFβ1 and BMP4 strongly induce the epicardial EMT in these cells. Therefore, we used TGFβ1 as an inducer of the epicardial EMT in subsequent experiments.

We also examined changes in protein expression during the TGFβ1-induced epicardial EMT by western blot (Figure S5). Consistent with the results shown in Figure S4B, Vcam1 was decreased and N-cadherin was increased by treatment with a lower concentration of TGFβ1. In addition, Tbx18 expression was not changed but Wt1 expression was decreased by TGFβ1 treatment (Figure S5).

**Figure 3:**
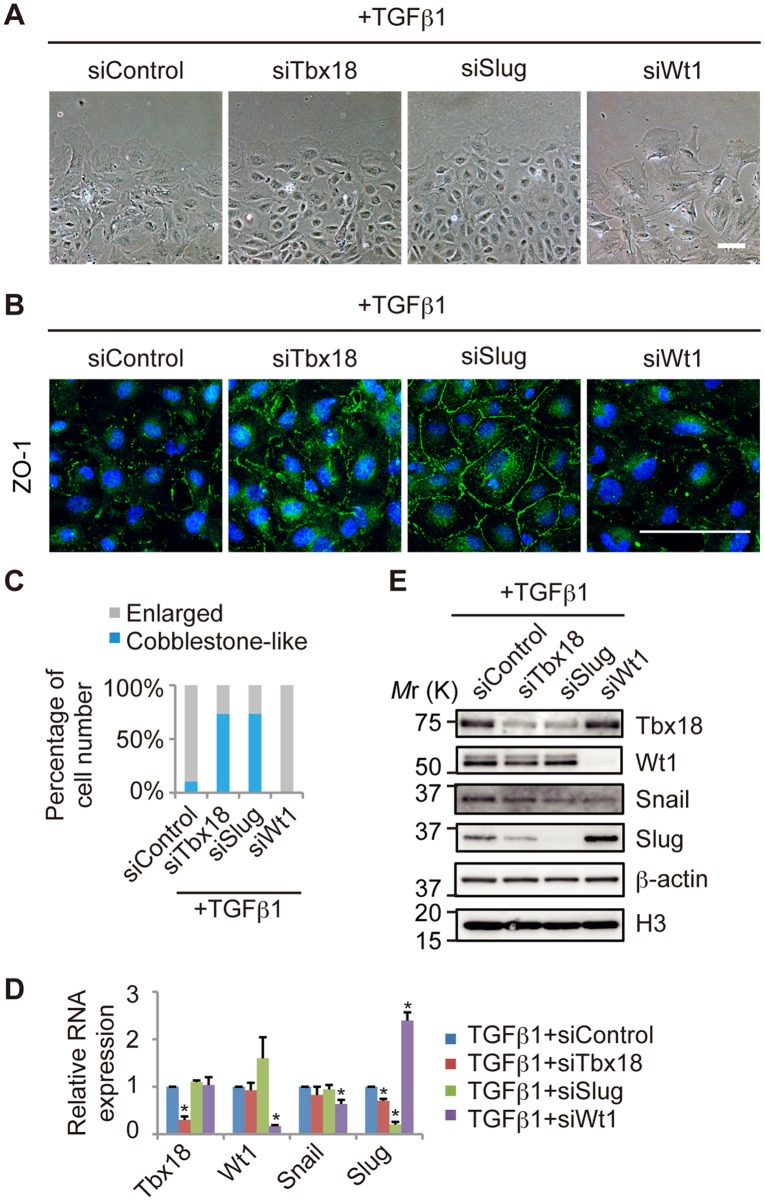
The epicardial EMT induced by TGFβ1 is inhibited by knockdown of Tbx18 or Slug. (A) Representative images of epicardial cells treated with TGFβ1 and transfected with control siRNA (siControl) or siRNA directed against Tbx18 (siTbx18), Slug (siSlug) or Wt1 (siWt1). (B) Immunostaining for ZO-1 (green) and DAPI nuclear staining (blue) of primary epicardial cells transfected with siRNAs. (C) Percentage of cells categorized as “Enlarged” or “Cobblestone-like,” based on cellular morphology. (D) The relative mRNA expression of *Tbx18*, *Wt1*, *Snail* and *Slug* by real-time PCR analysis in TGFβ1-treated epicardial cells transfected with siControl, siTbx18, siSlug or siWt1 (n = 3; *P<0.05 vs. siControl). The results were normalized to *Gapdh* expression, and the relative expression level is given as a ratio to the TGFβ1+siControl. (E) Western blot performed with antibodies against Tbx18, Wt1, Snail and Slug. β-actin and histone H3 were used as loading controls. The data are presented as the mean ± SD. Scale bars: 100 µm.

We then performed knockdown experiments of Tbx18, Slug or Wt1 during the TGFβ1-induced epicardial EMT. TGFβ1 successfully induced the epicardial EMT in siControl cells under this experimental condition, as evidenced by cellular morphology and ZO-1 expression (Figure S6). Knockdown of Tbx18 or Slug (siSlug) attenuated the TGFβ1-induced morphological changes (Figure 3A). Slug knockdown restored ZO-1 localization at the cell-cell junction; however, the effect of Tbx18 knockdown was weaker than that of Slug knockdown, suggesting that Tbx18 only partially contributes to ZO-1 expression (Figure 3B). By contrast, Wt1 knockdown increased morphological changes, as evidenced by the increased number of cells with a spread shape and impaired cell-cell contacts (Figure 3A, 3B and 3C, and Figure S1B).

Importantly, Slug expression was decreased in Tbx18-knockdown cells and increased in Wt1-knockdown cells during the TGFβ1-induced epicardial EMT, consistent with the observed changes in cellular morphology (Figure 3D and 3E). By contrast, *Snail* mRNA expression was slightly decreased in Wt1-knockdown cells and was not significantly altered in Tbx18- or Slug-knockdown cells during the TGFβ1-induced mesenchymal transition (Figure 3D), suggesting that the functional downstream target of Tbx18 and Wt1 is Slug, not Snail. Tbx18 protein expression was also reduced following Slug knockdown, suggesting feedback regulation between Tbx18 and Slug expression, such that Slug stabilizes Tbx18 protein levels (Figure 3E). Tbx18 knockdown induced the upregulation of Tbx5, a member of the T-box family of transcription factors (Figure S7), suggesting that the lack of epicardial defects in Tbx18-knockout mice might be due to functional compensation by other T-box proteins. These data suggest that Slug and Tbx18 contribute to the TGFβ1-induced epicardial EMT and that Wt1 negatively regulates the epicardial EMT. Furthermore, it is likely that Slug functions as a downstream target of Tbx18 and Wt1.

### Knockdown of Tbx18 or Slug Inhibits the Epicardial EMT Induced by Wt1 Knockdown in Primary Epicardial Cells

**Figure 4:**
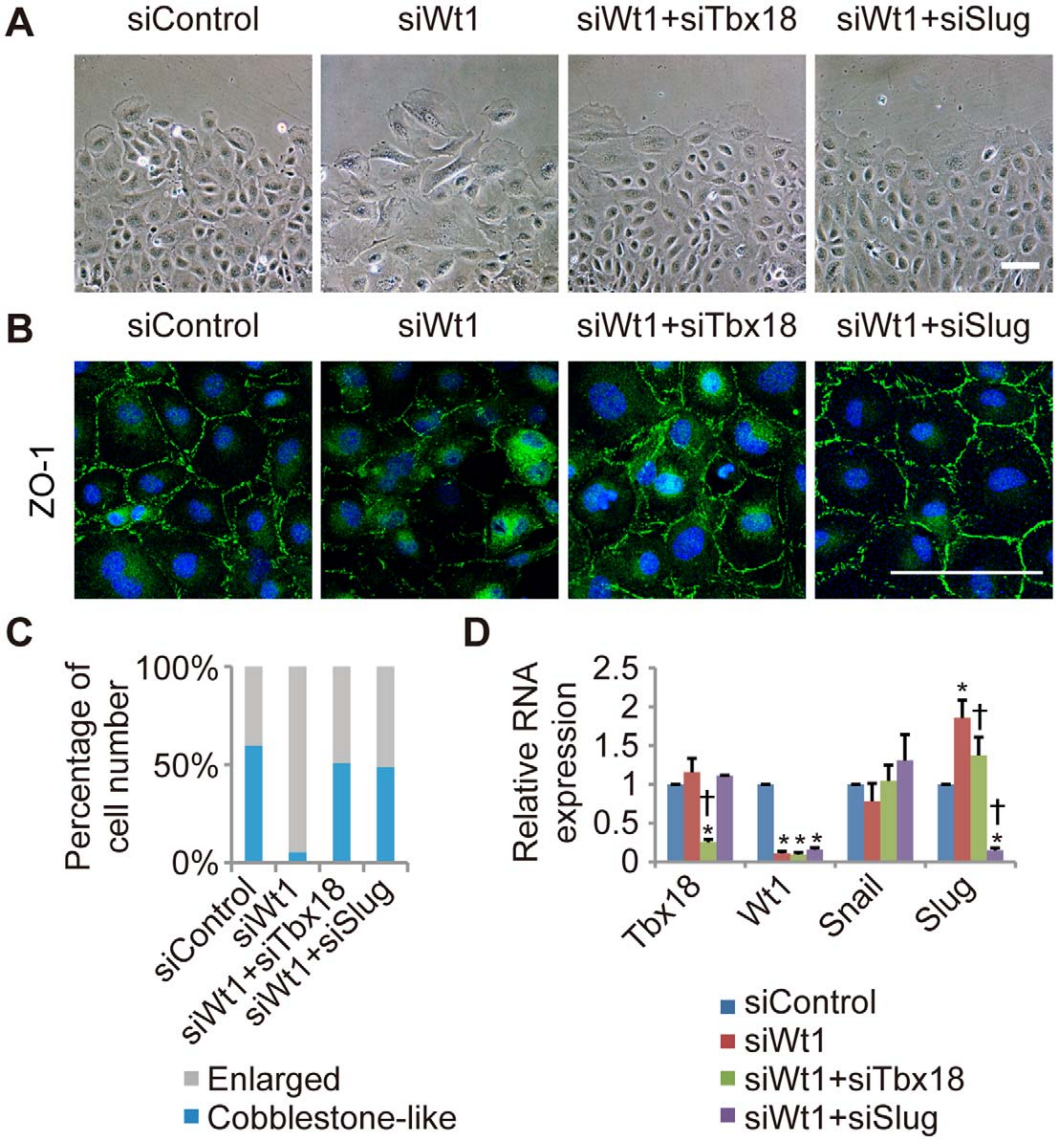
The epicardial EMT induced by Wt1 knockdown is inhibited by knockdown of Tbx18 or Slug. (A) Representative images of Wt1-knockdown epicardial cells co-transfected with siTbx18 or siSlug. Epicardial cells transfected with siControl or siWt1 were used as controls. (B) Immunostaining for ZO-1 (green) and DAPI nuclear staining (blue) of primary epicardial cells transfected with siRNA. (C) Percentage of cells categorized as “Enlarged” or “Cobblestone-like,” based on cellular morphology. (D) The relative mRNA expression of *Tbx18*, *Wt1* and *Slug* by real-time PCR in Wt1-knockdown epicardial cells co-transfected with siTbx18 or siSlug, in comparison to siControl-transfected epicardial cells (n = 3; *P<0.05 vs. siControl, ^†^ *P*<0.05 vs. siWt1). The results were normalized to *Gapdh* expression and the relative expression level is provided as a ratio to the siControl. The data are presented as the mean ± SD. Scale bars: 100 µm.

Because Slug is a key molecule in the epicardial EMT, we hypothesized that Tbx18 and Wt1 coordinately determine epicardial cell fate through the bi-directional regulation of Slug expression. To assess this hypothesis, we examined whether the mesenchymal transition induced by Wt1 knockdown is inhibited following knockdown of Tbx18 or Slug. Wt1-knockdown cells exhibited a mesenchymal morphology and decreased ZO-1 expression at the cell-cell junction, as shown in Figure 4A and 4B. These changes induced by Wt1 knockdown were inhibited by the co-transfection of an siRNA directed against Tbx18 or Slug (Figure 4A, 4B and 4C, and Figure S1C). Furthermore, the increase in Slug mRNA expression caused by Wt1 knockdown was reduced upon knockdown of Tbx18 (Figure 4D). Taken together, these results suggest that Tbx18 promotes the mesenchymal transition through the upregulation of *Slug,* and that Wt1 maintains the epicardial cell fate through the downregulation of *Slug*.

### Tbx18 and Wt1 Regulate Slug Expression in NMuMG-C7 Cells

**Figure 5:**
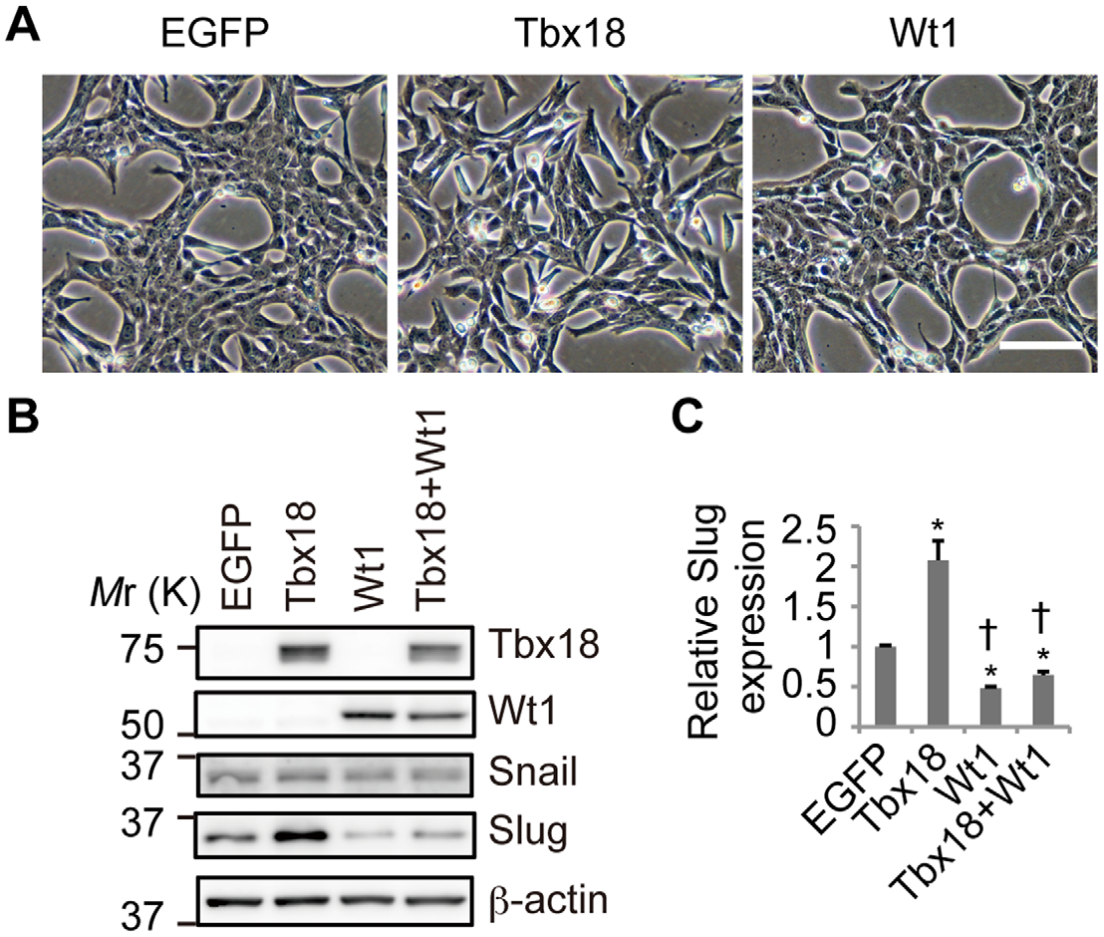
Tbx18 and Wt1 regulate Slug expression in NMuMG-C7 cells. (A) Representative images of NMuMG-C7 cells expressing EGFP, Tbx18, Wt1 or a combination of Tbx18 and Wt1. Scale bar: 100 µm. (B) Western blot analysis of transduced NMuMG-C7 cell lines with antibodies against Tbx18, Wt1, Snail and Slug. β-actin was used as a loading control. (C) The relative mRNA expression of *Slug* in transduced NMuMG-C7 cell lines. The results were normalized to *Gapdh* expression, and the relative expression is provided as a ratio to EGFP transduced cells. The data are presented as the mean ± SD; n = 3; *P<0.05 vs. EGFP transduced cells, ^†^ *P*<0.0001 vs. Tbx18 transduced cells.

To further investigate the molecular functions of Tbx18 and Wt1, overexpression experiments were performed. Because our results raise the possibility that Tbx18 and Wt1 have opposing functions, we used NMuMG-C7 cells, which do not express endogenous Tbx18 or Wt1. NMuMG-C7 cells were derived using the limiting dilution technique from NMuMG cells [38]. NMuMG-C7 cell lines that overexpress Tbx18 or Wt1 were established by retroviral transduction. As shown in Figure 5A, Wt1-expressing cells did not exhibit any changes in cell-cell adhesion or epithelial morphology when compared to the EGFP-expressing control cells. By contrast, Tbx18-expressing cells exhibited impaired cell-cell interactions and a fibroblastic morphology (Figure 5A).

The stable expression of Tbx18 or Wt1 in these cell lines was confirmed by western blot (Figure 5B). Compared to the control cells, Slug expression was increased in Tbx18-expressing cells and decreased in Wt1-expressing cells, consistent with the observed morphological phenotypes of these cells (Figure 5B and 5C). The increase in Slug expression that was triggered by the Tbx18 transduction was suppressed by Wt1 co-transduction, suggesting that Tbx18 and Wt1 coordinately regulate *Slug* expression (Figure 5B and 5C). Snail expression was not altered in these stable cell lines (Figure 5B), suggesting that Snail is not a potential target of Tbx18 or Wt1. As shown in Figure S8A and S8B, Tbx18-expressing cells exhibited an increase in cell migration compared to control cells. Based on the quantification of the mRNA levels of EMT-related molecules in Tbx18-expressing cells by real-time PCR, the expression of the epithelial adhesion molecule *E-cadherin* was decreased, while the expression levels of mesenchymal genes such as *Fibronectin*, *αSMA* and *Vimentin* were increased (Figure S8C). Together, these results suggest that Tbx18 increases Slug expression, while Wt1 decreases Slug expression in NMuMG-C7 cells that do not express endogenous Tbx18 or Wt1.

### Tbx18 and Wt1 Bind to the Slug Promoter Region and Regulate Slug Promoter Activity

**Figure 6:**
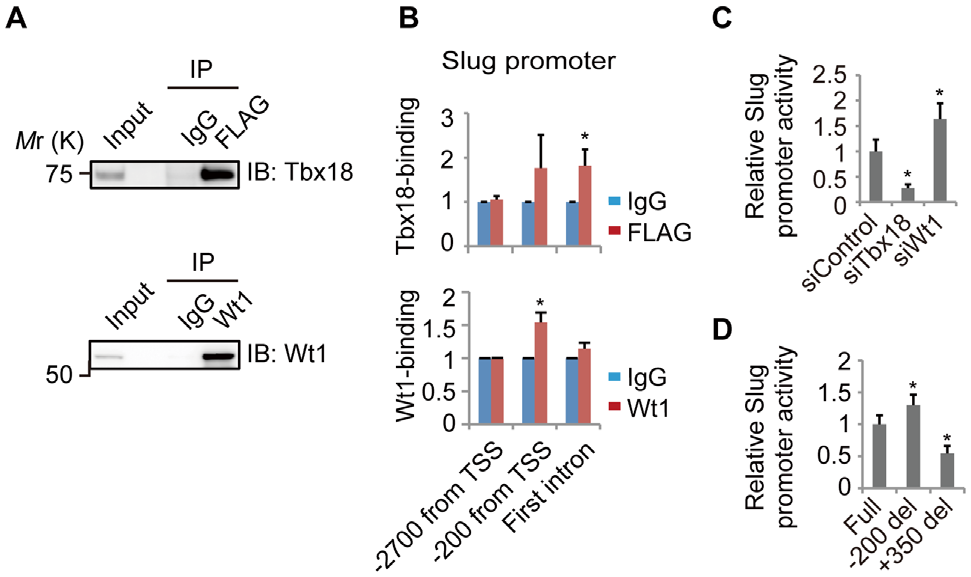
Tbx18 and Wt1 are bound to the Slug promoter region and regulate the activity of the Slug promoter. (A) Immunoblot performed with an anti-Tbx18 antibody and an anti-Wt1 antibody. Tbx18 was immunoprecipitated with an anti-FLAG antibody (FLAG) or control IgG (IgG) in NMuMG-C7 cells expressing 3×FLAG-tagged Tbx18, and Wt1 was immunoprecipitated with an anti-Wt1 antibody (Wt1) or control IgG (IgG) in NMuMG-C7 cells expressing Wt1. (B) Direct binding of Tbx18 or Wt1 near the transcription start site (TSS) of the Slug gene in NMuMG-C7 cells, as assessed by ChIP. DNA fragments co-precipitated with Tbx18 or Wt1 were quantified by real-time PCR. The data are presented as the mean ± SD; n = 3; *P<0.05 vs. control IgG. (C) The relative luciferase activity of a reporter construct carrying the *Slug* promoter in primary epicardial cells. The data are provided as ratios to siControl and are presented as the mean ± SD; n = 4; *P<0.01 vs. siControl. (D) The relative luciferase activity of a reporter construct carrying the *Slug* promoter (Full) or the *Slug* promoter lacking the region around −200 from the TSS (−200 del) or +350 from the TSS (+350 del) in primary epicardial cells. The data are provided as ratios to Full and are presented as the mean ± SD; n = 4; *P<0.05 vs. Full.

According to our experiments with primary epicardial cells and NMuMG-C7 cells, Slug is a potential downstream target of Tbx18 and Wt1. Thus, we investigated the direct binding of these transcription factors to the *Slug* promoter region in NMuMG-C7 cells by ChIP. In NMuMG-C7 cells expressing 3×FLAG-tagged Tbx18, we observed Tbx18 protein bound to the first intron of the *Slug* gene (Figure 6A and 6B). In addition, in NMuMG-C7 cells expressing Wt1, we detected Wt1 bound near the transcription start site (TSS) (Figure 6A and 6B).

Furthermore, we examined whether the binding of Tbx18 or Wt1 is important for *Slug* promoter activity in primary epicardial cells. As shown in Figure 6C, *Slug* promoter activity was decreased as a result of Tbx18 knockdown but was increased following Wt1 knockdown. In addition, the deletion of the −200 region from TSS, which our results suggest is the Wt1 binding site, from the *Slug* promoter, resulted in an increase in luciferase activity, while the deletion of the +350 region from TSS, which our results suggest is the site of Tbx18 localization, from the *Slug* promoter, resulted in a decrease in luciferase activity (Figure 6D). These findings suggest that Tbx18 and Wt1 bind to different *Slug* promoter regions and directly regulate the level of *Slug* expression level.

## Discussion

In this study, we determined that 2 transcription factors, Tbx18 and Wt1, play important roles in the regulation of the epicardial EMT. In primary epicardial cells, knockdown of Wt1 resulted in the loss of the epicardial phenotype, with an increase in cellular migration and enhanced mesenchymal morphology, whereas knockdown of Tbx18 attenuated the epicardial EMT induced by TGFβ1 or Wt1 knockdown. Slug appears to be a common downstream target that is upregulated by Tbx18 and downregulated by Wt1, and Tbx18 and Wt1 were observed to bind the *Slug* promoter region and regulate *Slug* promoter activity.

T-box family proteins are essential for many aspects of heart development, including the mesenchymal transition of endocardial cells [44]–[46]. Tbx18 is a member of the T-box family and is expressed in the epicardium and mesenchymal cells near the venous pole in the heart as well as in the somites, the ureteral mesenchyme and the otic mesenchyme during development [14]. The loss of Tbx18 leads to the delayed differentiation of the sinus horn myocardium [16], [17], skeletal malformation [47], kidney defects [48] and deafness [49]. However, defects in the epicardium have not been reported in Tbx18-knockout mice, and transgenic mice overexpressing Tbx18 in epicardium-derived cells do not exhibit any epicardial cell defects [18].

Our results indicate that Tbx18 maintains *Slug* expression in primary epicardial cells and that the knockdown of either Tbx18 or Slug inhibits the epicardial EMT induced by TGFβ1 or Wt1 knockdown. Although Tbx18-knockout mice did not exhibit any defects in the epicardial EMT, we speculate that there is a compensatory mechanism that replaces the function of Tbx18. We observed that the expression of Tbx5, which is known to exist in both the developing epicardium and myocardium [50], was increased following Tbx18 knockdown in primary epicardial cells (Figure S7), implying that the lack of Tbx18 may be compensated for by other T-box transcription factors in Tbx18-knockout mice. Tbx5 might perform functions that are similar to those of Tbx18, as Tbx5 is also expressed in the epicardium and belongs to the T-box protein family. We do not have data to explain why Tbx5 compensates for Tbx18 function in knockout mice but not in knockdown primary epicardial cells. However, because Tbx18 knockdown is transient, the compensatory effect might be weaker or incomplete in Tbx18-knockdown cells compared with Tbx18-knockout mice, which lack Tbx18 during all developmental stages. In addition, we realized that the effect of Tbx18 knockdown in the epicardial EMT was weaker than that of Wt1 knockdown. Tbx18 knockdown did not completely attenuate ZO-1 expression, even though the morphological changes were clearly restored.

The inhibitory effect of other molecules might explain why epicardial overexpression of Tbx18 does not affect the epicardial EMT in mice [18]. We observed that Tbx18 was necessary for the epicardial EMT induced by Wt1 knockdown in primary epicardial cells; however, the epicardial EMT was not induced in untreated primary epicardial cells that express both Tbx18 and Wt1. Therefore, we speculate that Wt1 may attenuate the effects of Tbx18-overexpression in the epicardium. Tbx18 might be necessary but not sufficient for the epicardial EMT to occur.

Wt1 contributes to heart development through the regulation of retinoic acid signaling. *Wt1*-null mice die at mid-gestation and exhibit defects in the formation of several organs [19], including a characteristic thinning of the myocardium that is similar to the phenotype of retinoic acid receptor (*Rxra*) mutant mice [51], [52]. Because myocardial cells do not express Wt1, this cardiac muscle phenotype is believed to be dependent on Wt1-regulated epicardial paracrine signals [20], [53]. Further research into *Wt1*-null embryos has shown that coelomic cells lining the liver also display decreased Raldh2 expression concomitant with liver hypoplasia [54]. Guadix et al. have indicated that Wt1 critically regulates epicardial retinoic acid signaling through direct activation of the *Raldh2* gene and have identified a role for Wt1 in the regulation of the morphogen receptors that are involved in the proliferation, migration and differentiation of epicardial and epicardium-derived cells [23]. In another report, von Gise et al. demonstrated that Wt1-knockout hearts exhibited diminished proliferation of the compact myocardium and impaired coronary plexus formation and that the Wt1-knockout epicardium failed to undergo the epicardial EMT [22]. They concluded that Wt1 regulates not only the epicardial EMT but also heart development through canonical Wnt, non-canonical Wnt and retinoic acid signaling pathways.

Martinez-Estrada et al. demonstrated that the epicardial cells of *Wt1* conditional-knockout mice (Gata5-Cre) exhibited an increase in E-cadherin expression and a decrease in Snail expression [21]. They also examined the function of Wt1 in the EMT of embryonic stem cells. Finally, these researchers demonstrated that Wt1 is required for the epicardial EMT through transcriptional control of Snail and E-cadherin. They performed immunostaining of E-cadherin *in vivo*; however, von Gise et al. claimed that E-cadherin was not observed in the epicardium of either wild-type or Wt1-knockout mice by immunohistochemistry [22]. We also could not detect the immnofluorescent signal of E-cadherin in Wt1-knockdown primary epicardial cells, and we observed a decrease, not an increase, in E-cadherin in Wt1-knockdown primary epicardial cells by western blot. Therefore, E-cadherin is likely not upregulated by the loss of Wt1 in the epicardium. In addition, a recent study demonstrated that the epicardial EMT occurs independently of Snail expression [55]. Based on these facts, we speculate that Wt1 might regulate the epicardial EMT through another downstream molecule.

In this study, we demonstrated that Wt1 is necessary for the maintenance of epicardial properties through the downregulation of Slug expression. Wt1-knockdown primary epicardial cells exhibit an enlarged morphology, with decreased ZO-1 expression at the cell-cell junctions and increased migratory activity, suggesting that Wt1 knockdown induced the epicardial EMT in primary epicardial cells. Slug expression was increased by Wt1 knockdown, and the mesenchymal transition induced by Wt1 knockdown was attenuated by Slug knockdown. These results suggest that Wt1 inhibits epicardial EMT by suppressing Slug expression. The differences between our results and those of previous *in vivo* studies might be due to differences in the experimental procedures. Previous studies analyzed Wt1-knockout mice, but we performed knockdown experiments using primary epicardial cells. In Wt1-knockout mice, Wt1 is not expressed during any developmental stage. The complete loss of Wt1 influences not only the epicardial EMT but also the development of the whole heart, including the thinning of the myocardium and discontinuous epicardium [22]. In these mice, it is possible that the epicardium is not mature, which might cause the decrease in migrated epicardial cells. Indeed, epicardial thinning in Wt1-knockout mice has been previously described [22]. Thus, Wt1 is required for whole heart development, including events in early heart development such as epicardial specification or maturation and myocardial growth as well as the epicardial EMT.

In knockdown experiments using primary epicardial cells, the lack of Wt1 is transient, and heart development proceeds normally until the knockdown experiments. Our knockdown experiments may suggest that Wt1 is required for the maintenance of epicardial cell properties after normal epicardial maturation. In agreement with our results, Bax et al. reported that the mesenchymal transition was induced by Wt1 knockdown in human adult epicardial cells [24]. Their observations suggest that Wt1 is necessary for maintaining either epithelial or epicardial morphology in cultured cells. Bax et al. demonstrate that Snail is increased after Wt1 knockdown; by contrast, we observed upregulation of Slug but not Snail. We speculate that the character of the epicardial cells used by Bax et al. is slightly different from the character of our epicardial cells. Bax et al. obtained human epicardium from adult hearts by biopsy and expanded the cells in FGF-containing medium. The adult epicardium usually does not express Wt1, but these cultured cells exhibited Wt1 expression, which was confirmed by immunostaining.

In this study, we confirmed that Tbx18 and Wt1 regulate Slug expression in primary epicardial cells and NMuMG-C7 cells, although the mechanism by which Snail expression is regulated remains unclear. In a previous report, von Gise et al. demonstrated that the expression of Slug mRNA tends to be reduced in Wt1-knockout mice; however, there is no significant difference when these mice are compared with control mice [22]. von Gise et al. also mentioned that Wt1-knockout mice exhibited an undeveloped epicardium; therefore, Slug expression could be decreased by the defective maturation of the epicardium. We also observed a slight downregulation of Snail in Wt1-knockdown epicardial cells, consistent with a previous report suggesting that Wt1 is required for the epicardial EMT due to its regulation of Snail [21]. However, the epicardial EMT was not dependent on Snail expression in our primary cells, as supported by another recent study [55]. These results suggest that Wt1 is required for epicardial maintenance rather than the mesenchymal transition by suppressing Slug expression. Indeed, the knockdown of Slug attenuated the epicardial EMT that had been induced by Wt1 knockdown.

Although the functions of Tbx18 and Wt1 were clearly demonstrated using primary epicardial cells in this study, there are experimental limitations. The primary epicardial cells that were used in this study are derived from a specific developmental stage (E12.5), the Tbx18 or Wt1 knockdown is transient, and the cultured epicardial cells lack an interaction with the myocardium. Therefore, our *in vitro* experimental system may reflect one phase of the epicardial EMT in the developing heart but not the effects of the complete loss of Tbx18 or Wt1 during all developmental stages.

During development and after injury, epicardial cells express both Tbx18 and Wt1 and have the potential to undergo the epicardial EMT; however, this does not occur in unprimed animals [14], [15], [28], [29], [56]. These ‘active’ epicardial cells localize to the outer surface of the heart and exhibit epithelial characteristics but maintain high motility and the potential to transform into mesenchymal cells. The plastic properties of the ‘active’ epicardium may be achieved by the simultaneous expression of Tbx18 and Wt1, two functionally opposing transcription factors. We have demonstrated that Wt1 knockdown induces the upregulation of Slug and the mesenchymal transition in primary epicardial cells; both of these changes were attenuated by Tbx18 knockdown. These results suggest that Tbx18 and Wt1 control the epicardial-mesenchymal balance by fine-tuning Slug expression coordinately, but not individually.

Collectively, our results indicate the importance of Tbx18 and Wt1, as well as their downstream target Slug, during the epicardial EMT. Wt1 knockdown induces the epicardial EMT, and the knockdown of Tbx18 or Slug attenuates the epicardial EMT induced by TGFβ1 treatment or Wt1 knockdown. Our results provide new insights into the molecular mechanisms regulating the epicardial EMT and might be beneficial for the *ex* and *in vivo* manipulation of epicardial cells under various conditions.

## Supporting Information

Figure S1 **Representative images of primary epicardial cells used for cell counts.** We categorized the cells into 2 groups (epicardial and mesenchymal) based on cellular morphology using the criteria described in the Materials and Methods section. The counted areas (yellow squares) were chosen so that the distance from the margin of each colony was 820 µm. (A) Representative images of cells that were used for Figure 3B. (B) Representative images of cells that were used for Figure 4B. (C) Representative images of cells that were used for Figure 5B. Scale bars: 200 µm.
(TIF)

Figure S2 **The proliferation of primary epicardial cells is not affected by Tbx18 or Wt1 knockdown.** Immunostaining for BrdU (green) in primary epicardial cells transfected with control siRNA (siControl) or an siRNA directed against Tbx18 (siTbx18) or Wt1 (siWt1). Scale bar: 100 µm.
(TIF)

Figure S3 **Cellular morphology and mRNA expression in primary epicardial cells transfected with other siRNAs.** (A) Representative images of primary epicardial cells that were transfected with control siRNA (siControl), siTbx18-2 or siWt1-2. (B) The relative mRNA expression levels in the primary epicardial cells described in A. The results were normalized to *Gapdh* expression, and the relative expression level is provided as a ratio to the siControl. The data are presented as the mean ± SD; n = 3; *P<0.01 vs. siControl. Scale bar: 100 µm.
(TIF)

Figure S4 **Primary epicardial cells undergo the epicardial EMT after TGFβ1- or BMP4-treatment.** (A) Representative images of primary epicardial cells after 3 days of treatment with recombinant soluble factors (TGFβ1 at 10 ng/ml; BMP4, BMP2, FGF2, FGF9, VEGF and PDGF-BB at 100 ng/ml; retinoic acid at 1 mM). (B) The relative mRNA expression level of epicardial adhesion molecules (*Integrin α4* and *Vcam1*) and a mesenchymal adhesion molecule (*N-cadherin*) in primary epicardial cells treated with TGFβ1 (10 ng/ml), BMP4 (100 ng/ml) or BMP2 (100 ng/ml) (n = 3; *P<0.01 vs. non-treated cells). The results were normalized to *Gapdh* expression, and the relative expression level is shown as a ratio to the no-treatment control. The data are presented as the mean ± SD. Scale bar: 200 µm.
(TIF)

Figure S5 **Protein expression in primary epicardial cells with or without TGFβ1 treatment.** Western blot showing the expression levels of Tbx18, Wt1, Vcam1 and N-cadherin in primary epicardial cells with or without TGFβ1 treatment (2 days, 1 ng/ml). β-actin and histone H3 were used as loading controls.
(TIF)

Figure S6 **Primary epicardial cells undergo the epicardial EMT after 1 day of treatment with TGFβ1.** (A) Representative images of primary epicardial cells after 1 day of treatment with TGFβ1 at 1 ng/ml. (B) Immunostaining for ZO-1 (green) and DAPI nuclear staining (blue) of primary epicardial cells in (A). Scale bars: 100 µm.
(TIF)

Figure S7 **Relative Tbx5 mRNA expression levels in primary epicardial cells transfected with control siRNA (siControl) or an siRNA directed against Tbx18 (siTbx18).** The results were normalized to *Gapdh* expression, and the relative expression level is provided as a ratio to the siControl. The data are presented as the mean ± SD; n = 3; *P<0.01 vs. siControl.
(TIF)

Figure S8 **Tbx18-induced EMT in NMuMG-C7 cells.** (A) Migration assay using a Boyden chamber. Scale bar: 100 µm. (B) The number of migrated cells was quantified. The data are presented as the mean ± SD; n = 16; *P<0.001 vs. EGFP control. (C) The relative mRNA expression levels of an epithelial molecule (*E-cadherin*) and mesenchymal molecules (*Fibronectin*, *αSMA*, *Vimentin*) were quantified by real-time PCR. The results were normalized to *Gapdh* expression, and the relative expression level is provided as a ratio to EGFP transduced cells. The data are presented as the mean ± SD; n = 3; *P<0.01 vs. EGFP control.
(TIF)

Table S1 Primers used in this study.
(DOCX)
