# Supplementary figures and images for: The Transcription Factors Tbx18 and Wt1 Control the Epicardial Epithelial-Mesenchymal Transition through Bi-Directional Regulation of Slug in Murine Primary Epicardial Cells
Source: PLoS One. 2013 Feb 28;8(2):e57829. doi: 10.1371/journal.pone.0057829 (PMC3585213; doi:10.1371/journal.pone.0057829)

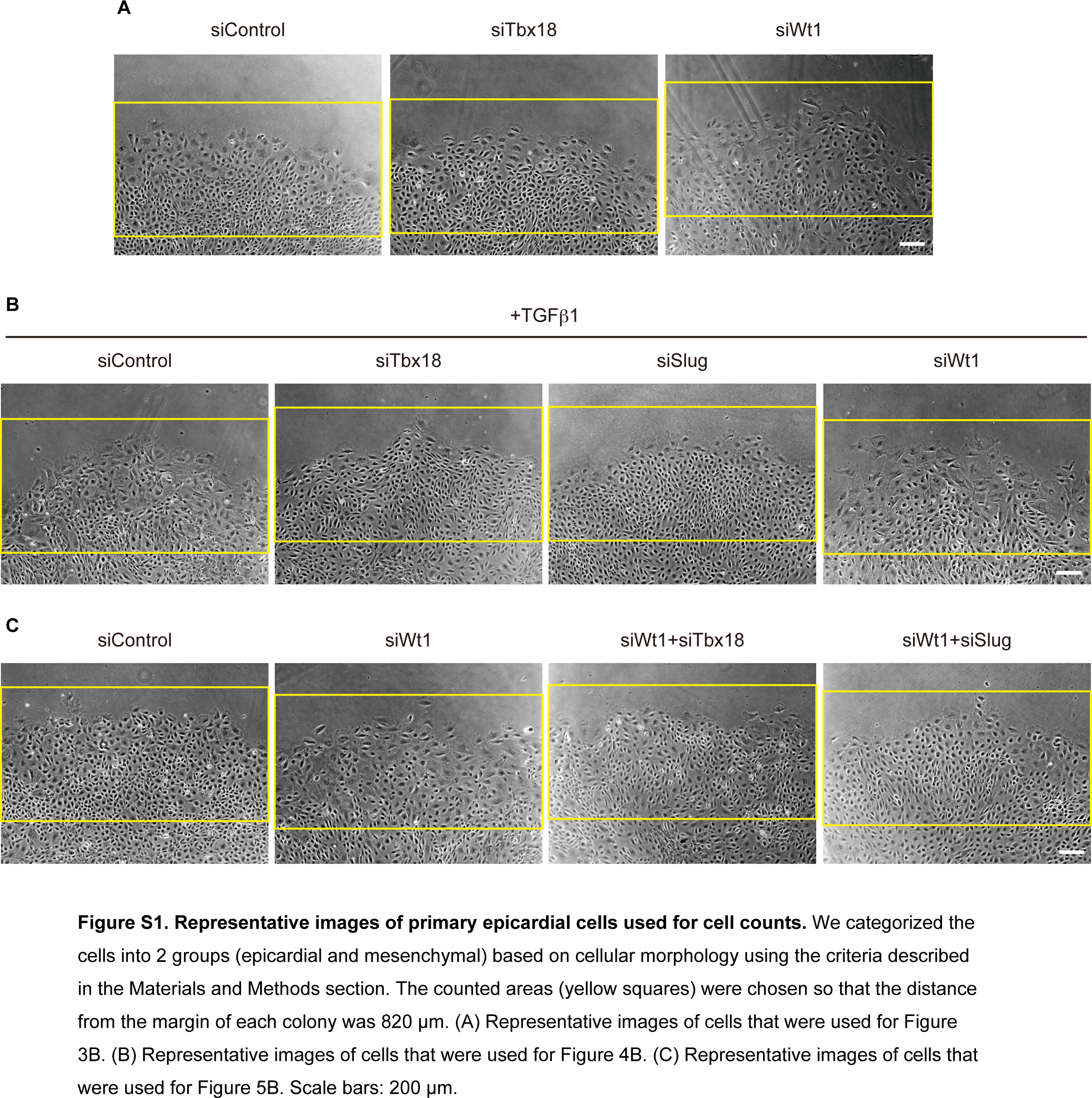

Supplement: Figure S1 — Representative images of primary epicardial cells used for cell counts. We categorized the cells into 2 groups (epicardial and mesenchymal) based on cellular morphology using the criteria described in the Materials and Methods section. The counted areas (yellow squares) were chosen so that the distance from the margin of each colony was 820 µm. (A) Representative images of cells that were used for Figure 3B. (B) Representative images of cells that were used for Figure 4B. (C) Representative images of cells that were used for Figure 5B. Scale bars: 200 µm. (TIF) [file pone.0057829.s001.tif]

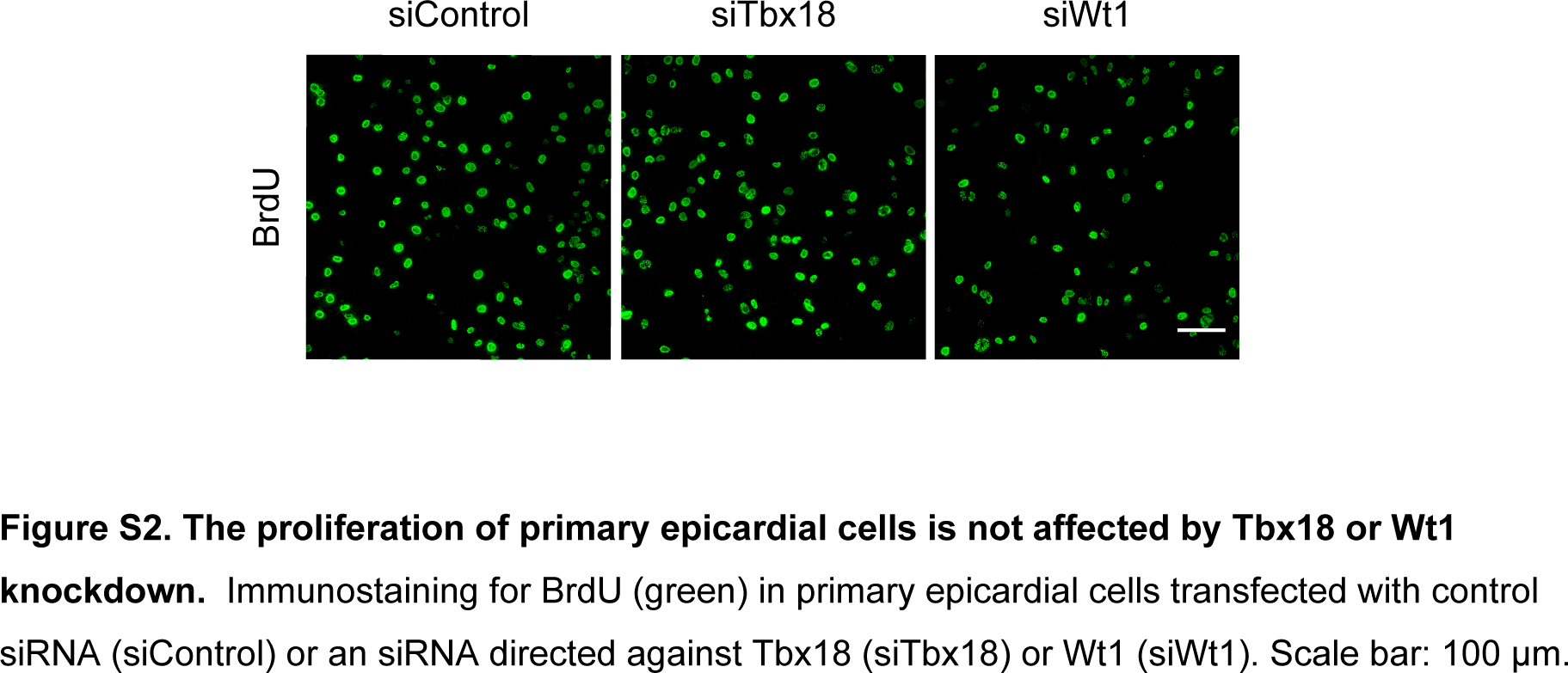

Supplement: Figure S2 — The proliferation of primary epicardial cells is not affected by Tbx18 or Wt1 knockdown. Immunostaining for BrdU (green) in primary epicardial cells transfected with control siRNA (siControl) or an siRNA directed against Tbx18 (siTbx18) or Wt1 (siWt1). Scale bar: 100 µm. (TIF) [file pone.0057829.s002.tif]

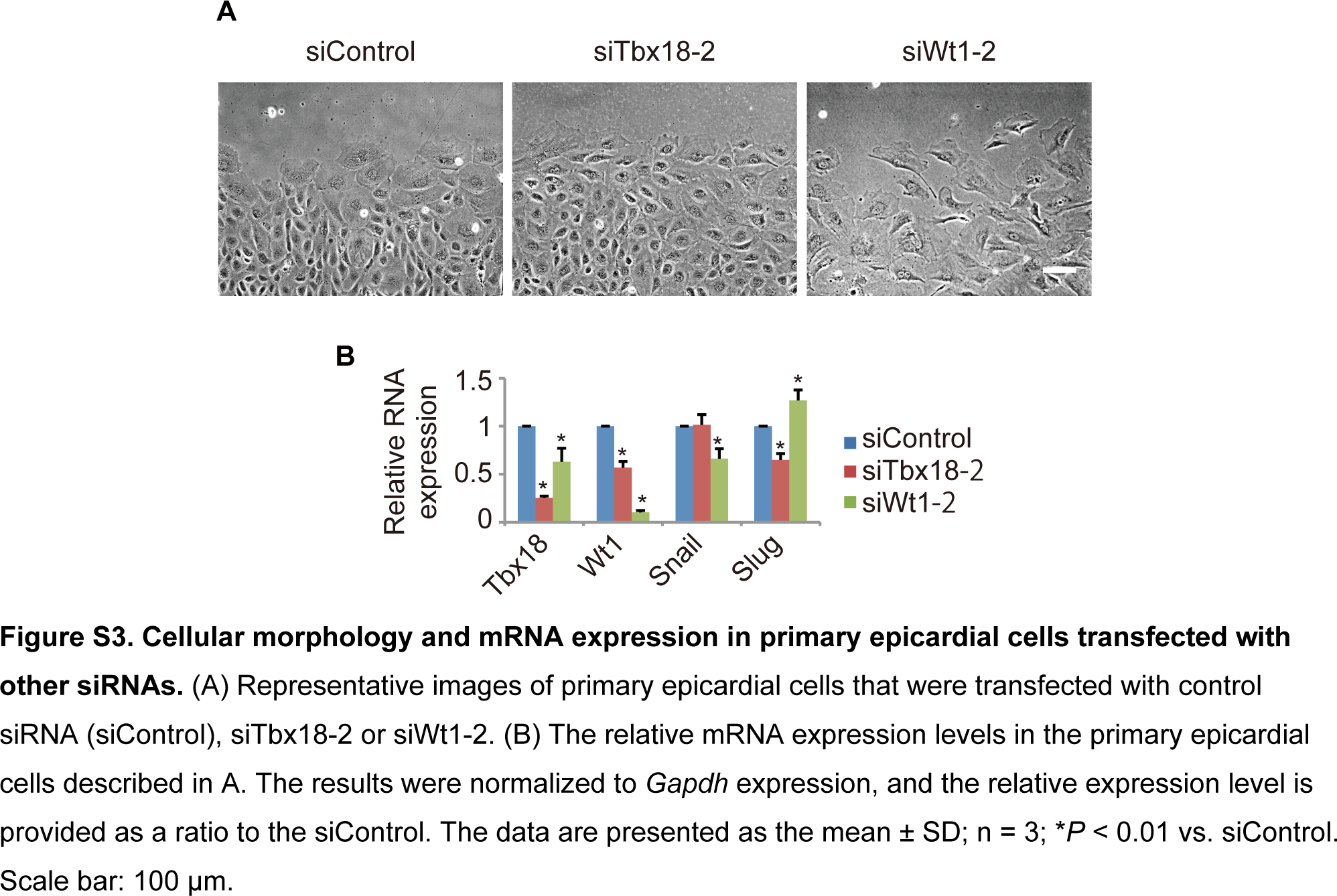

Supplement: Figure S3 — Cellular morphology and mRNA expression in primary epicardial cells transfected with other siRNAs. (A) Representative images of primary epicardial cells that were transfected with control siRNA (siControl), siTbx18-2 or siWt1-2. (B) The relative mRNA expression levels in the primary epicardial cells described in A. The results were normalized to Gapdh expression, and the relative expression level is provided as a ratio to the siControl. The data are presented as the mean ± SD; n = 3; *P<0.01 vs. siControl. Scale bar: 100 µm. (TIF) [file pone.0057829.s003.tif]

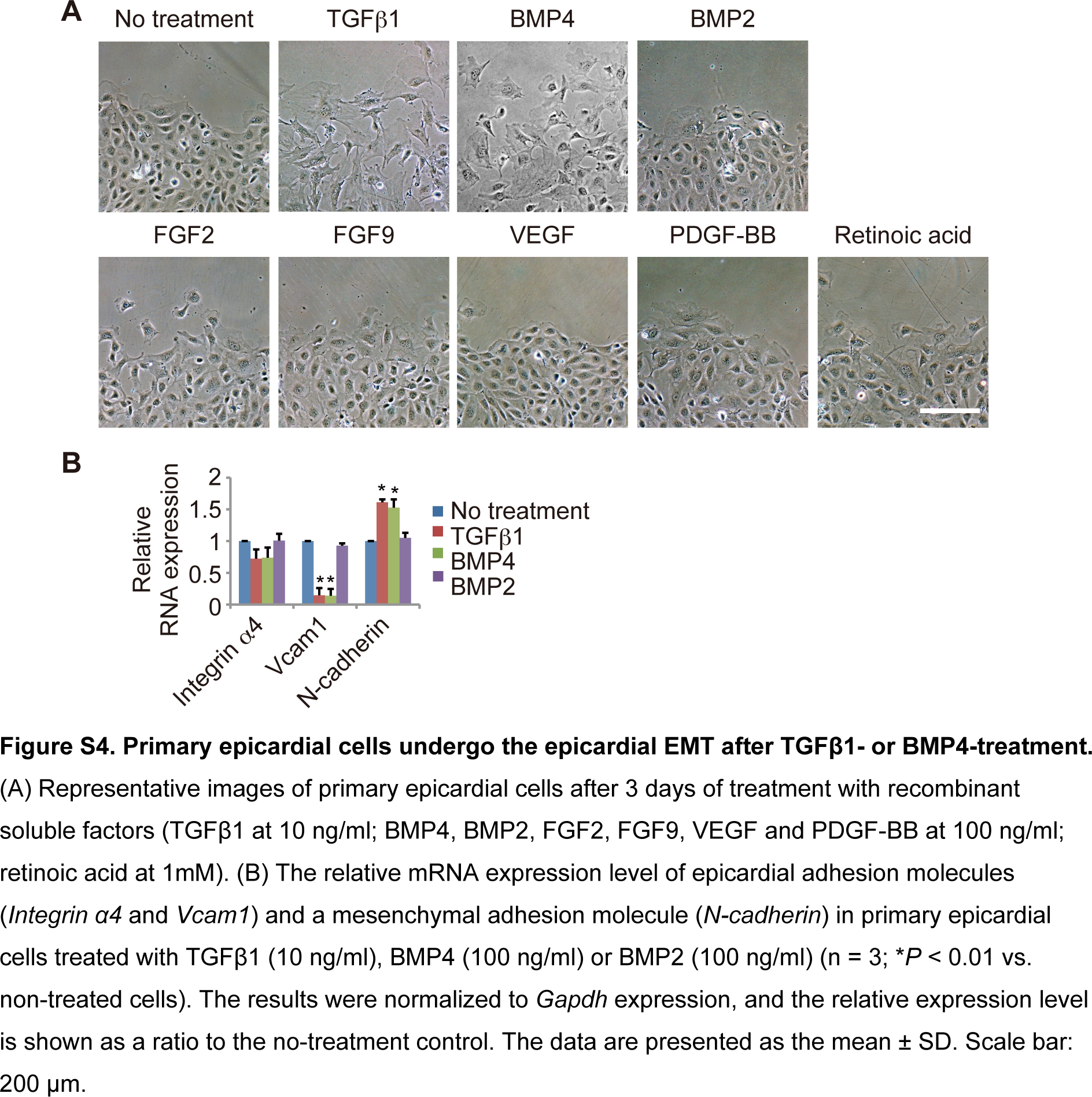

Supplement: Figure S4 — Primary epicardial cells undergo the epicardial EMT after TGFβ1- or BMP4-treatment. (A) Representative images of primary epicardial cells after 3 days of treatment with recombinant soluble factors (TGFβ1 at 10 ng/ml; BMP4, BMP2, FGF2, FGF9, VEGF and PDGF-BB at 100 ng/ml; retinoic acid at 1 mM). (B) The relative mRNA expression level of epicardial adhesion molecules (Integrin α4 and Vcam1) and a mesenchymal adhesion molecule (N-cadherin) in primary epicardial cells treated with TGFβ1 (10 ng/ml), BMP4 (100 ng/ml) or BMP2 (100 ng/ml) (n = 3; *P<0.01 vs. non-treated cells). The results were normalized to Gapdh expression, and the relative expression level is shown as a ratio to the no-treatment control. The data are presented as the mean ± SD. Scale bar: 200 µm. (TIF) [file pone.0057829.s004.tif]

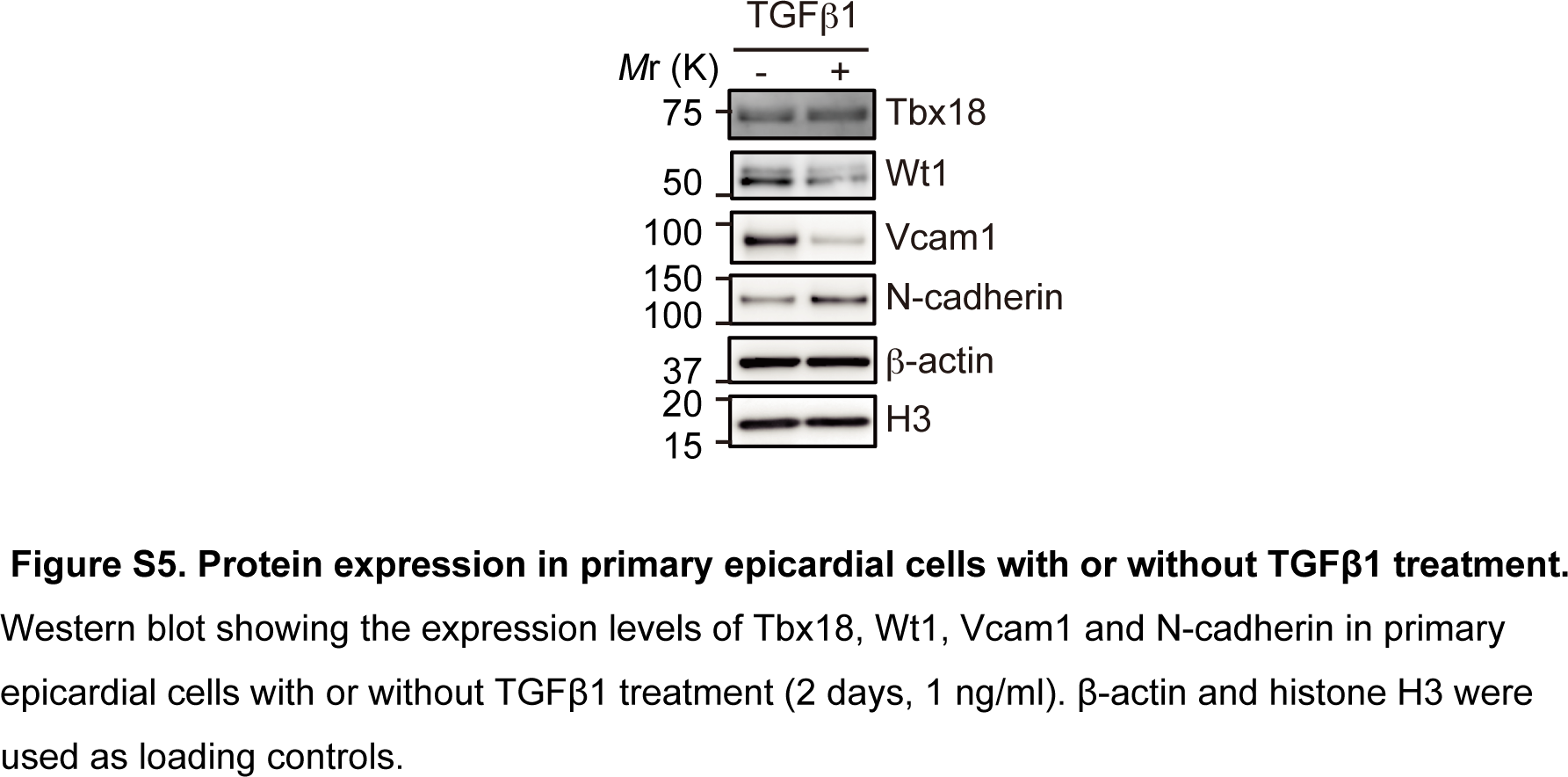

Supplement: Figure S5 — Protein expression in primary epicardial cells with or without TGFβ1 treatment. Western blot showing the expression levels of Tbx18, Wt1, Vcam1 and N-cadherin in primary epicardial cells with or without TGFβ1 treatment (2 days, 1 ng/ml). β-actin and histone H3 were used as loading controls. (TIF) [file pone.0057829.s005.tif]

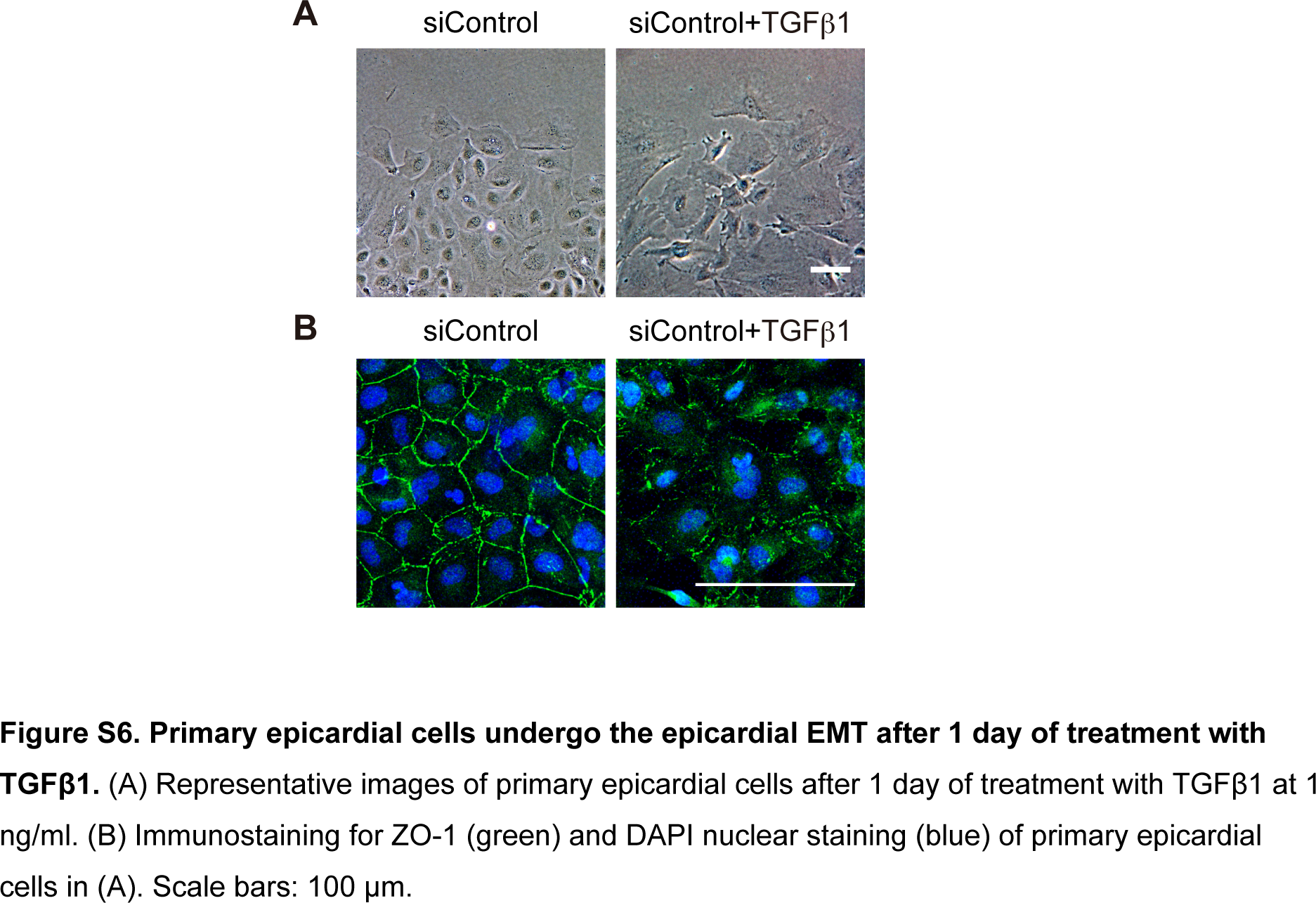

Supplement: Figure S6 — Primary epicardial cells undergo the epicardial EMT after 1 day of treatment with TGFβ1. (A) Representative images of primary epicardial cells after 1 day of treatment with TGFβ1 at 1 ng/ml. (B) Immunostaining for ZO-1 (green) and DAPI nuclear staining (blue) of primary epicardial cells in (A). Scale bars: 100 µm. (TIF) [file pone.0057829.s006.tif]

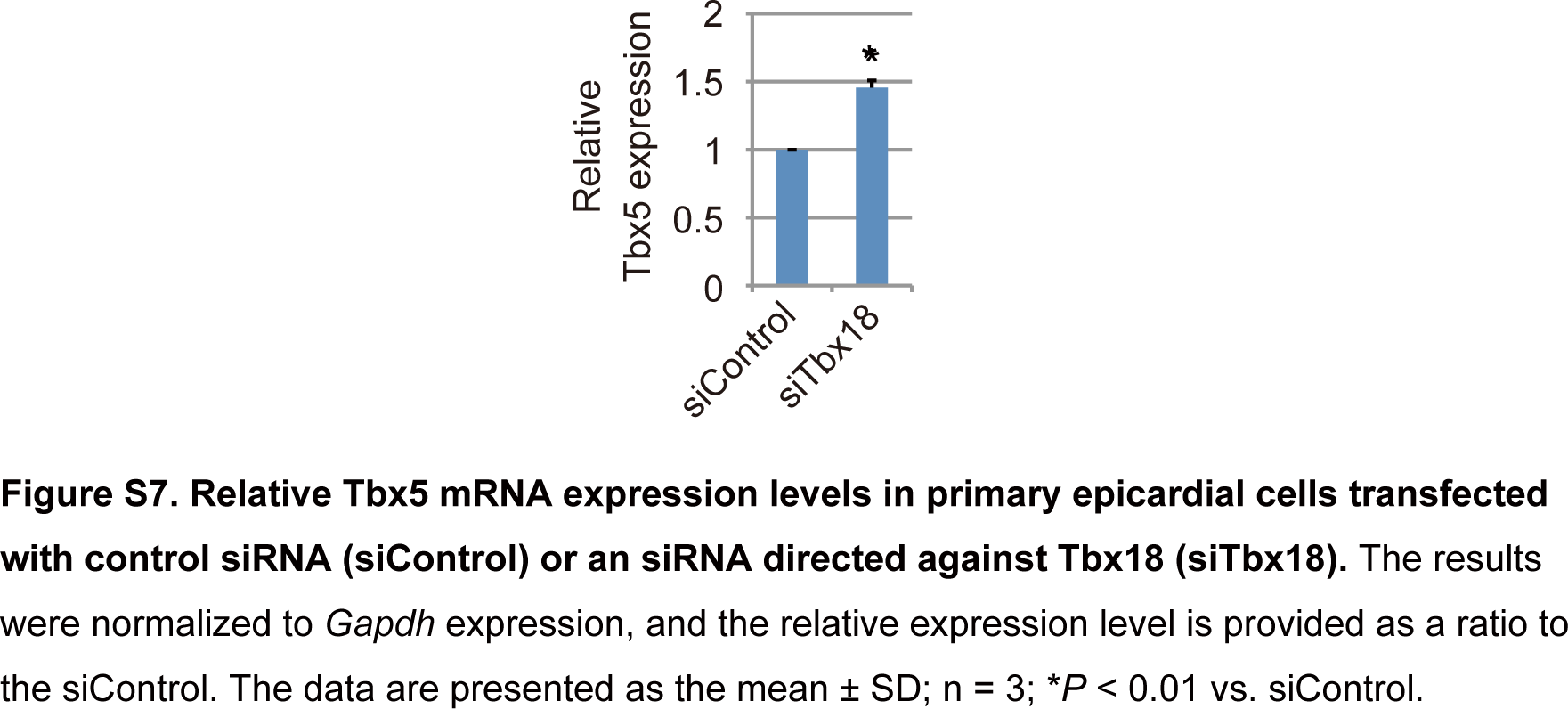

Supplement: Figure S7 — Relative Tbx5 mRNA expression levels in primary epicardial cells transfected with control siRNA (siControl) or an siRNA directed against Tbx18 (siTbx18). The results were normalized to Gapdh expression, and the relative expression level is provided as a ratio to the siControl. The data are presented as the mean ± SD; n = 3; *P<0.01 vs. siControl. (TIF) [file pone.0057829.s007.tif]

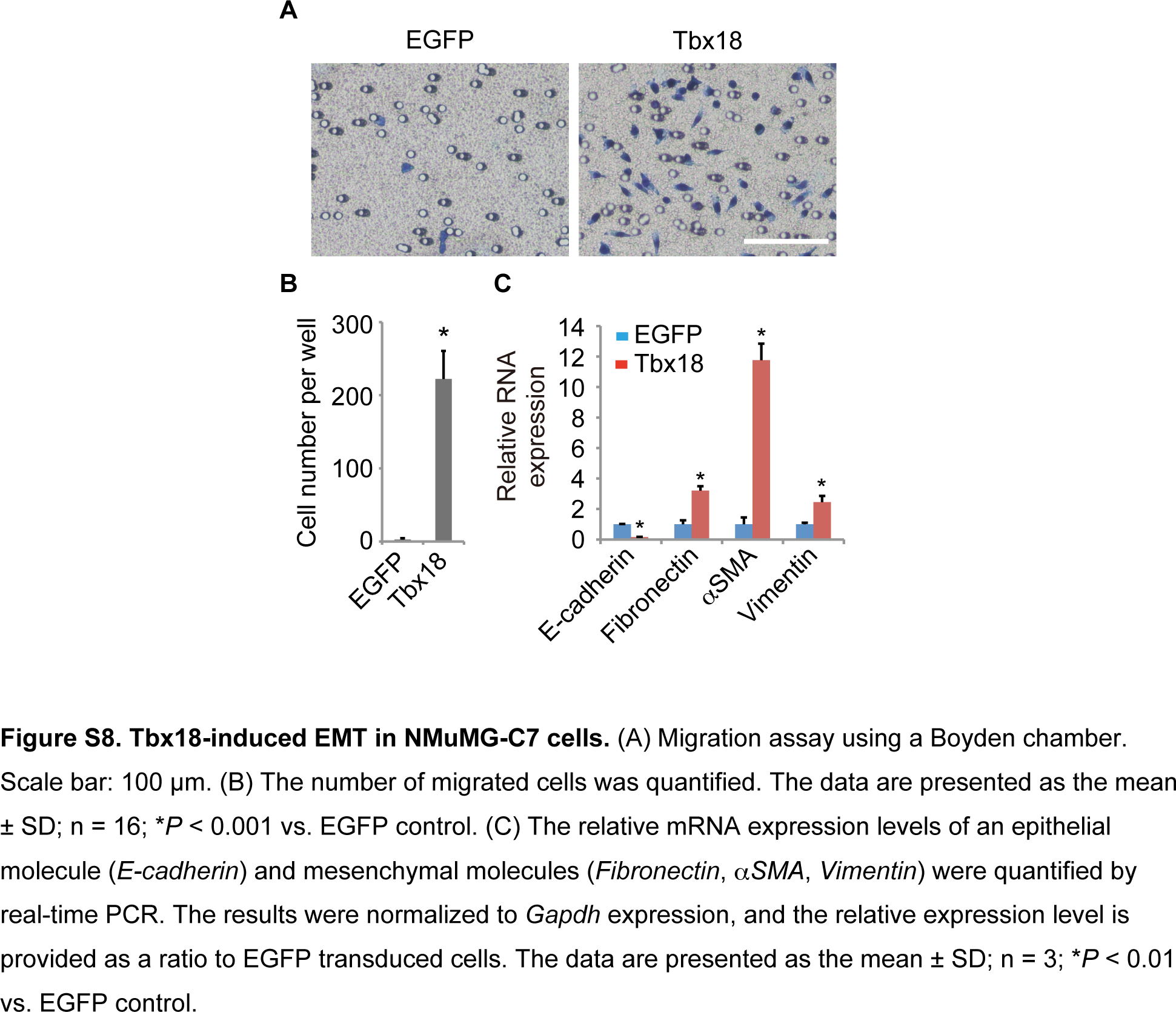

Supplement: Figure S8 — Tbx18-induced EMT in NMuMG-C7 cells. (A) Migration assay using a Boyden chamber. Scale bar: 100 µm. (B) The number of migrated cells was quantified. The data are presented as the mean ± SD; n = 16; *P<0.001 vs. EGFP control. (C) The relative mRNA expression levels of an epithelial molecule (E-cadherin) and mesenchymal molecules (Fibronectin, αSMA, Vimentin) were quantified by real-time PCR. The results were normalized to Gapdh expression, and the relative expression level is provided as a ratio to EGFP transduced cells. The data are presented as the mean ± SD; n = 3; *P<0.01 vs. EGFP control. (TIF) [file pone.0057829.s008.tif]
