# Supplementary material for: The Transcription Factors Tbx18 and Wt1 Control the Epicardial Epithelial-Mesenchymal Transition through Bi-Directional Regulation of Slug in Murine Primary Epicardial Cells
Source: PLoS One. 2013 Feb 28;8(2):e57829. doi: 10.1371/journal.pone.0057829 (PMC3585213; doi:10.1371/journal.pone.0057829)
Supplement: Table S1 — Primers used in this study. (DOCX) [file pone.0057829.s009.docx]

**Table S1.** Primers used in this study

| Integrin α4 | F; 5’-accagacctgcgaacagc-3’  R; 5’-cccccagccactggttat-3’ | qRT-PCR for Integrin α4 |
| --- | --- | --- |
| Vcam1 | F; 5’-tggtgaaatggaatctgaacc-3’  R; 5’-cccagatggtggtttcctt-3’ | qRT-PCR for Vcam1 |
| N-cadherin | F; 5’-tgacccagaagatgatgtaagaga-3’  R; 5’-tggagctggctcaagtcata-3’ | qRT-PCR for N-cadherin |
| Fibronectin | F; 5’-tgcacgatgatatggagagc-3’  R; 5’-tgggtgtcacctgactgaac-3’ | qRT-PCR for Fibronectin |
| αSMA | F; 5’-ccagcaccatgaagatcaag-3’  R; 5’-tggaaggtagacagcgaagc-3’ | qRT-PCR for αSMA |
| Vimentin | F; 5’-cgaggagagcaggatttctc-3’  R; 5’-ggaagtgactccaggttagtttct-3’ | qRT-PCR for Vimentin |
| Has2 | F; 5’-gttggaggtgttggaggaga-3’  R; 5’-catccagtatctcacgctgct-3’ | qRT-PCR for Has2 |
| Snail | F; 5’-gtctgcacgacctgtggaa-3’  R; 5’-caggagaatggcttctcacc-3’ | qRT-PCR for Snail |
| Slug | F; 5’-tgcaagatctgtggcaagg-3’  R; 5’-cagtgagggcaagagaaagg-3’ | qRT-PCR for Slug |
| -2700 from TSS | F; 5’-TCAGGAAGCCTTGTGGGAAA-3’  R; 5’-TCTCATGGGACCCTGGAAAG-3’ | ChIP for -2700 region from TSS in Slug promoter |
| -200 from TSS | F; 5’-GGTCACCTAGCGGAAACACG-3’  R; 5’-TGCTCATGGCATTTCAGTGG-3’ | ChIP for -200 region from TSS in Slug promoter |
| First intron | F; 5’-TGCCAAAACTGTCCTTGCAG-3’  R; 5’-CACGTCCGTGCACTCCTTTT-3’ | ChIP for first intron in Slug promoter |
| H1foo | F; 5’-AGGCTAGCAGTAGTCTGGATCAG-3’  R; 5’-ACTGTGTCCTACCTACCTGACGAG-3’ | ChIP for H1Foo |
